# SR 4233 (tirapazamine): a new anticancer drug exploiting hypoxia in solid tumours.

**DOI:** 10.1038/bjc.1993.220

**Published:** 1993-06

**Authors:** J. M. Brown

**Affiliations:** Department of Radiation Oncology, Stanford University, California 94305.

## Abstract

SR 4233 (3-amino-1,2,4-benzotriazine 1,4-dioxide, WIN 59075, tirapazamine) is the lead compound in a new class of bioreductive anticancer drugs, the benzotriazine di-N-oxides. It is currently undergoing Phase I clinical testing. The preferential tumour cell killing of SR 4233 is a result of its high specific toxicity to cells at low oxygen tensions. Such hypoxic cells are a common feature of solid tumours, but not normal tissues, and are resistant to cancer therapies including radiation and some anticancer drugs. The killing of these tumour cells by SR 4233, particularly when given on multiple occasions, can increase total tumour cell killing by fractionated irradiation by several orders of magnitude without increasing toxicity to surrounding normal tissues. Topics covered in this review include the rationale for developing a hypoxic cytotoxic agent, the cytotoxicity of SR 4233 as a function of oxygen concentration, the mechanism of action of the drug and its intracellular target and the in vivo evidence that the drug may be useful as an adjunct both to radiotherapy and chemotherapy. Finally, the major unanswered questions on the drug are outlined.


Br. J. Cancer (1993), 67, 1163–1170 © Macmillan Press Ltd., 1993

REVIEW

### SR 4233 (Tirapazamine): a new anticancer drug exploiting hypoxia in solid tumours

J.M. Brown

*Department of Radiation Oncology, Stanford University, Stanford, California 94305, USA.*

**Summary** SR 4233 (3-amino-1,2,4-benzotriazine 1,4-dioxide, WIN 59075, tirapazamine) is the lead compound in a new class of bioreductive anticancer drugs, the benzotriazine di-N-oxides. It is currently undergoing Phase I clinical testing. The preferential tumour cell killing of SR 4233 is a result of its high specific toxicity to cells at low oxygen tensions. Such hypoxic cells are a common feature of solid tumours, but not normal tissues, and are resistant to cancer therapies including radiation and some anticancer drugs. The killing of these tumour cells by SR 4233, particularly when given on multiple occasions, can increase total tumour cell killing by fractionated irradiation by several orders of magnitude without increasing toxicity to surrounding normal tissues. Topics covered in this review include the rationale for developing a hypoxic cytotoxic agent, the cytotoxicity of SR 4233 as a function of oxygen concentration, the mechanism of action of the drug and its intracellular target and the *in vivo* evidence that the drug may be useful as an adjunct both to radiotherapy and chemotherapy. Finally, the major unanswered questions on the drug are outlined.

### Why develop a hypoxia-selective cytotoxic drug?

A key strategy in cancer treatment is to try to exploit some intrinsic difference between normal and malignant tissues. One such difference is that a large proportion of solid tumours contain cells at lower levels of oxygenation than occurs in normal tissues. Such hypoxia in tumours is found in the vast majority of transplanted rodent tumours (Moulder & Rockwell, 1987) and in human tumours xenografts in immunodeficient mice (Rockwell & Moulder, 1990). There is also compelling evidence from a variety of techniques that hypoxic cells are also present in human solid tumours (Hockel *et al.*, 1991; Mueller-Klieser *et al.*, 1981; Vaupel *et al.*, 1991). However, the lower oxygenation level of many solid tumours compared to normal tissues has only recently been seen as therapeutically exploitable. Indeed, because of the resistance of hypoxic cells to killing by ionising radiation, their presence in tumours would be expected to adversely affect cure rates in radiotherapy, and there is considerable evidence that this is the case for several types of malignancies (Bush *et al.*, 1978; Gatenby *et al.*, 1988; Henk & Smith, 1977; Overgaard *et al.*, 1986). Preclinical studies suggest that hypoxic cells may also be refractory to certain chemotherapeutic drugs (Kennedy, 1987; Sartorelli, 1988; Tannock & Guttman, 1981).

Three general strategies have been developed to overcome the perceived problem of the hypoxic cells in solid tumours. First, efforts have been made to increase tumour oxygenation using a variety of means, the most recent of which is to combine nicotinamide with carbogen (95% $O_2$ plus 5% $CO_2$) (Chaplin *et al.*, 1991; Kjellen *et al.*, 1991). The second approach has been to develop chemical sensitisers to preferentially increase the radiation sensitivity of the hypoxic cells (Adams, 1981; Brown, 1989). A third approach has been to develop hypoxic cell cytotoxins, agents that can selectively kill hypoxic cells (Kennedy, 1987; Rockwell *et al.*, 1982; Sartorelli, 1988; Zeman *et al.*, 1986).

For each of these three general approaches, the rationale is to overcome the *problem* of hypoxic cells in tumours. However, it has recently been proposed that the presence of hypoxic cells may be an advantage to the therapy of tumours (Brown & Koong, 1991). This would only be the case, however, under certain conditions, namely:

(1) An agent with high selective toxicity for hypoxic cells is combined with one with selective toxicity to aerobic cells (such as ionising radiation).
(2) The dynamics of the tumour are such that the percent of hypoxic and aerobic cells in the tumour at the time of each of the treatments with the combination of agents is roughly the same as at the time of the first treatment (i.e., that both reoxygenation and 'rehypoxiation' (Brown & Lemmon, 1992) occur following each treatment).

This is the rationale for developing an agent with high selective toxicity for hypoxic cells. Although this biological rationale was not appreciated at the time, the paradigm for a bioreductive drug was suggested many years ago when Sartorelli and colleagues (Lin *et al.*, 1972) proposed that the low oxygen levels of solid tumours might be conducive to bioreductive metabolism of a drug to generate a compound more toxic than the parent compound. This concept of bioreductive activation has been extensively reviewed (e.g. Kennedy, 1987; Lin *et al.*, 1976; Moore, 1977), and three general classes of such agents are now known. First, the quinone antibiotics of which mitomycin C is the prototype drug (Rockwell *et al.*, 1982; Sartorelli, 1988). Second, the nitroimidazoles, although developed as radiation sensitisers, show preferential toxicity to hypoxic cells which is high in the case of the dual function agent, RSU 1069 (Stratford & Stephens, 1989; Stratford *et al.*, 1986). The third class of bioreductive cytotoxic drugs are the benzotriazine di-N-oxides of which SR 4233 (3-amino-1,2,4-benzotriazine 1,4-dioxide, WIN 59075, tirapazamine) is the prototype compound (Zeman *et al.*, 1986). SR 4233 is presently in Phase 1 clinical testing and, in fact, is the first drug to be introduced into the clinic purely as a bioreductive cytotoxic agent. How best to exploit such an agent and whether this, or other bioreductive drugs to follow, can turn hypoxic cells into a therapeutic advantage in solid tumours have yet to be answered.

### *Preferential cytotoxicity of SR 4233 for mammalian cells* in vitro

In the first report of the activity of SR 4233, Zeman and colleagues presented data on seven different cell lines of hamster, mouse and human origin and found differential hypoxic cytotoxicity for all the cell lines with a hypoxic cytotoxicity ratio (concentration of drug in air divided by concentration of drug in hypoxia to produce the same level

Received 30 November 1992; and in revised form 1 February 1993.

of cell killing) of approximately 25 to 200 for the different lines. Suggested by their data was the possibility that human lines may have somewhat lower differential toxicities than rodent lines. Figure 1 shows pooled data from several experiments with three different cell lines showing a similar broad range of hypoxic cytotoxicity ratios for these lines. Values in the range of 50 to 200 have, in general, been observed by most investigators (Adams *et al.*, 1992; Costa *et al.*, 1989; Stratford & Stephens, 1989), although it has also been reported that certain cell lines with repair deficiencies have somewhat lower differential cytotoxicities (Keohane *et al.*, 1990).

Despite the variability in differential hypoxic cytotoxicity for different cell lines shown for SR 4233, values obtained from different investigators are consistently higher than those obtained for the quinone antibiotic mitomycin C, for which values of only 1 to 5 are found (Fracasso & Sartorelli, 1986; Keohane *et al.*, 1990; Stratford & Stephens, 1989), and for the 2-nitroimidazole misonidazole for which values of five to 15 are typical (Stratford & Stephens, 1989; Taylor & Rauth, 1978). However, comparable high values are often, though not universally, reported for the dual function nitroimidazole, RSU 1069 (Adams *et al.*, 1992; Roizin-Towle *et al.*, 1990; Stratford & Stephens, 1989).

**Figure 1** Survival of three cell lines to various concentrations of SR 4233 for a 90 min exposure at 37°C under aerobic or hypoxic conditions. The cell lines and their tumours of origin were: SCCVII, a mouse squamous cell carcinoma; HT1080, a human fibrosarcoma; and A549, a human lung carcinoma. The results show the pooled data from 3–6 independent experiments for each cell line with the lines drawn by eye. The dashed lines on the panels for the two human tumour cell lines are a reproduction of the curves from the SCCVII cells. Shown on the lower panel is the method of calculating the hypoxic cytotoxicity ratio (HCR). The method of achieving hypoxia was a modification of that described by Koch which allowed rapid gas equilibration of the cells attached to glass petri dishes (Koch, 1984). The average oxygen concentration in the medium was measured using a Clark type electrode (Koch, 1991) and was between 100 and 200 p.p.m. during the 90 min exposure.

How much of a differential is necessary? One answer would be that the differential should be large enough that substantial killing of hypoxic cells occurs with little or no killing of aerobic cells. This would be ideal, since it would mean that the dose of an agent (such as radiation) that kills well oxygenated normal cells would not have to be modified when the bioreductive drug was given. For such a criterion a factor of 10 or more would probably be adequate. Another answer is that the differential should be as large as possible because it is the cytotoxicity to aerobic cells which is likely to produce systemic toxicity, and this needs to be kept to a minimum. Thus, a drug design criterion would be that the hypoxic cytotoxicity ratio should be at least 10 and preferably larger. To date, of the bioreductive drugs in or nearing clinical testing, only the benzotriazine di-N-oxide, SR 4233, and the bifunctional nitroimidazole, RSU 1069 (or its prodrug, RB 6145 (Jenkins *et al.*, 1990)), fit this criterion.

*Oxygen requirement for preferential cytotoxicity*

The profile of drug toxicity as a function of oxygen concentration is an important, but often overlooked, characteristic of bioreductive cytotoxic agents. Just how low an oxygen level is needed to produce substantial differential hypoxic cytotoxicity will determine how big a subpopulation in a tumour is killed by the agent. Too low an oxygen level and few cells in the tumour will be differentially susceptible. Too high an oxygen level and various normal tissue cells will be killed by the drug. Mitomycin C is an example of a drug for which the hypoxia level is too stringent for maximum efficacy, since extremely low levels of oxygen (of less than 0.01% or 100 parts per million) are required to obtain maximum sensitivity (Marshall & Rauth, 1986). This is considerably lower than the oxygen level needed for maximum sensitivity for misonidazole (Koch, 1990; Mulcahy, 1984) and leaves cells at intermediate oxygen concentrations resistant to both mitomycin C and to radiation (Marshall & Rauth, 1986).

Figure 2 shows calculated profiles of cell killing by X-rays, SR 4233 and RSU 1069 as a function of oxygen concentration (Koch, personal communication, October 1992). These calculations are based on a comprehensive study of the survival of V79 cells exposed to SR 4233, to RSU 1069 and to misonidazole as a function of oxygen concentration (Koch, 1993). Although the aerobic/hypoxic ratios (HCR's) of SR 4233 and RSU 1069 in this study were similar when cells equilibrated with air were compared with those under ex-

**Figure 2** Calculations of the cell killing by a fixed dose of X-rays, SR 4233, RSU 1069 and the combination of X-rays with each drug as a function of oxygen concentration (Koch, personal communication, 1992). These calculation are based on measurements of the cytotoxicity of various bioreductive drugs (including RSU 1069 and SR 4233) to V79 cells *in vitro* as a function of oxygen concentration (Koch, 1993). The surviving fractions for the drug + radiation groups are the product of the independent survivals for drug (RSU 1069 or SR 4233) alone and for X-rays alone and therefore assume no interaction between the two agents. The dose of each agent has been chosen to produce a maximum level of cell killing of approximately $10^{-2}$ when given alone.

treme hypoxia, the profile at intermediate oxygen concentrations was quite different for the two drugs. Essentially, SR 4233 maintains its 'hypoxic cytotoxicity' at oxygen concentrations approximately 10-fold higher than those of RSU 1069. This results in the calculated additive killing produced by X-rays and SR 4233 being more uniform over the whole range of oxygen concentrations than for X-rays with RSU 1069. This is likely to be an advantage in tumours which often have oxygen concentrations spanning the range from extreme hypoxia to fully aerobic (Hockel *et al.*, 1991; Vaupel *et al.*, 1991).

### *Mechanism of hypoxic toxicity of SR 4233*

Several groups have investigated the mechanism for the selective hypoxic toxicity of SR 4233, and there is general agreement on the broad outlines of the mechanism. Figure 3 shows a diagram of the proposed mechanism for the selective cytotoxicity. It shows that the damaging species is an oxidising radical which is 'back-oxidised' to the parent drug in the presence of oxygen. This oxidising radical abstracts hydrogen from cellular targets leaving an oxidised target molecule along with the stable 2-electron reduction product, SR 4317.

The principle evidence for the above mechanism is listed below:

* When SR 4233 is added to mammalian cells under hypoxic, but not aerobic, conditions, cytotoxicity occurs as well as production of the metabolite, SR 4317. SR 4317, however, is toxic neither to hypoxic, nor aerobic, cells, despite the fact that it is taken up by the cells (Baker *et al.*, 1988; Costa *et al.*, 1989).
* The necessity for drug reduction to cause cytotoxicity is supported by the quantitative relationship between reduction rates and cytotoxicity for different cell lines and environmental conditions (Biedermann *et al.*, 1991; Costa *et al.*, 1989).
* The hypothesis that a free radical is responsible for cytotoxicity is supported by the finding that DMSO, a potent radical scavenger, substantially reduces hypoxic cytotoxicity (Brown, 1991).
* Definitive identification of the free radical has been obtained using electron spin resonance, with the unpaired electron being identified as primarily centred on the 1-nitrogen (Lloyd *et al.*, 1991). Evidence suggests that the radical anion is protonated to form a neutral oxidising radical capable of abstracting hydrogen from cellular targets (Baker *et al.*, 1988; Laderoute *et al.*, 1988). Also supporting this is the finding that the $V_{max}$ and $K_m$ for production of the radical (Lloyd *et al.*, 1991) are the same as the $V_{max}$ and $K_m$ for production of the 2-electron reduction product, SR 4317 (Walton & Workman, 1990).
* Under aerobic conditions, the superoxide radical has been identified (Lloyd *et al.*, 1991) as expected for an oxygen dependent futile cycling reaction as shown in Figure 3.

Although there is agreement that the first reductive step to the radical anion requires cellular enzymes, there is disagreement as to the principal enzyme involved. Using mouse liver microsomes as the enzyme source, Walton and co-workers (Walton *et al.*, 1992; Walton & Workman, 1990) identified cytochrome P450 as the major reductase in SR 4233 metabolism based on their finding of an approximately 80% inhibition of metabolism by the P450 inhibitor, carbon monoxide. Similarly, Wang and colleagues (Wang *et al.*, 1993) also identified cytochrome P450 as the major reductase using homogenates of a mouse and a human tumour cell line. On the other hand, Cahill and White and Lloyd and co-workers (Cahill & White, 1990; Lloyd *et al.*, 1991) found no inhibition of reduction of SR 4233 by rat liver microsomes using carbon monoxide and identified NADPH cytochrome P450 reductase as the major enzyme involved in drug reduction. Whether this disagreement reflects a difference between mouse and human on the one hand and rat on the other, or a technical problem with the carbon monoxide inhibition experiments, is not clear at present.

### *What is the target for cell killing?*

Various authors have shown that SR 4233 under hypoxic conditions produces both single- and double-stranded breaks in DNA (Laderoute *et al.*, 1988; Zeman & Brown, 1989). Data implicating DNA double-strand breaks as an important lesion in SR 4233 hypoxic cytotoxicity were obtained by Biedermann and colleagues (Biedermann *et al.*, 1991), who showed that two hamster cell lines, XR1 and V3, which are deficient in double-strand break rejoining and sensitive to X-irradiation, are also more sensitive to killing by SR 4233 than expected from their rates of metabolism of the drug under hypoxia.

More recently, definitive evidence that chromosome breaks can entirely account for cell killing by SR 4233 under hypoxia has been obtained for both hamster and human cells (Wang *et al.*, 1992, and unpublished). These investigators

**Figure 3** Schematic representation of the mechanism of cell killing by SR 4233 under hypoxic conditions. Cellular enzyme(s) (reductase[s]) reduce the drug (by adding a single electron), thereby producing a free radical which, after protonation, abstracts H from cellular molecules. In the case of DNA, this leads to single and double-strand breaks and to chromosome aberrations (which cause cell killing). In the presence of oxygen, the SR 4233 radical is oxidised back to the parent molecule (with the concomitant formation of $O_2^{\cdot -}$), thereby largely preventing the radical induced damage.

performed a quantitative comparison of the initial and the final number of chromosome breaks (using premature chromosome condensation) from equitoxic doses of X-irradiation and SR 4233 treatment. They showed that for equal cell killing, the *same* number of chromosome breaks were found for the two treatments after repair was complete. Since it is well known that chromosome breaks can entirely account for cell killing by ionising radiation (Cornforth & Bedford, 1987; Revell, 1983), it would appear that the same is true for SR 4233 exposure under hypoxia. Interestingly, however, these investigators found *fewer initial* chromosome breaks from SR 4233 treated cells than from X-irradiated cells at equal toxicity and showed that these breaks were less repairable than the X-ray produced breaks. A model to account for this finding is shown in Figure 4. It suggests that the breaks produced by SR 4233 are produced by high local concentrations of the activated radical as a consequence of the close proximity of an activating reductase. Such high local concentrations of radicals could produce highly damaged DNA similar to that produced by densely ionising radiation. Breaks such as these are less repairable than those produced by sparsely ionising radiation such as X-rays (Bedford & Goodhead, 1989; Prise *et al.*, 1987).

An implication of this work showing the importance of chromosome breaks, particularly if the model shown in Figure 4 is correct, is that identification of enzymatic reduction from whole cells or from microsomes may not identify the enzyme responsible for cytotoxicity, since this may reflect only a fraction of the total cellular metabolism. Relevant to this is the finding of Cahill and White that preparations of rat liver nuclei were able to metabolise SR 4233 to SR 4317, but at only 4% of the rate for rat liver microsomes (Cahill & White, 1990). Yet, this minor component of the reduction rate could be largely responsible for cytotoxicity.

To date, there are no published studies identifying either the mechansim of cell killing or the cellular target for cytotoxicity of SR 4233 under *aerobic* conditions. Such killing could be the result either of the parent SR 4233, the superoxide radical or the same SR 4233 radical as is responsible for hypoxic cytotoxicity. The cellular target responsible for cell killing by SR 4233 under aerobic conditions has not been identified. Studies of both of these are important because the systemic toxicity of the drug (assuming this is related to aerobic toxicity) may be the result of a different mechanism from that killing hypoxic tumour cells. If this were the case, it is possible that the tumour cell toxicity could be increased without changing the systemic toxicity or the systemic toxicity could be decreased without changing tumour cell toxicity, thereby increasing the therapeutic ratio.

### *Does SR 4233 preferentially kill hypoxic cells in tumours?*

The fact that SR 4233 preferentially kills hypoxic cells *in vitro* is no guarantee that it also preferentially kills hypoxic cells in solid tumours because of such problems as inadequate diffusion, too rapid metabolism or prohibitive systemic toxicity. Mitomycin C, for example, while demonstrating modest preferential killing of hypoxic cells *in vitro*, shows little or no preferential killing of hypoxic cells in mouse tumours (Rockwell & Kennedy, 1979). Zeman and colleagues investigated this question by combining SR 4233 with a single dose of irradiation given to mouse tumours (Zeman *et al.*, 1988). They found a roughly 20-fold increase of cell kill over that expected from the toxicity of radiation alone and that of SR 4233 alone. Although this is consistent with preferential killing of hypoxic cells, they also found that there was an interaction between the cytotoxicities of irradiation and SR 4233 under hypoxia which complicated the interpretation.

More definitive experiments have recently shown that the 'radiobiological' hypoxic fraction in SCCVII tumours (determined by the paired survival curve method) falls to approximately 8% of pretreatment levels by 1 h following SR 4233 injection (Kim & Brown, 1993). These and other experiments with fractionated irradiation (see below) strongly suggest that SR 4233 is preferentially cytotoxic to hypoxic cells in rodent solid tumours.

### *How should SR 4233 be used clinically?*

To date, there are no clinical studies with a drug which is specifically toxic to hypoxic cells. It might be argued that the clinical trial of mitomycin C plus radiotherapy (Weissberg *et al.*, 1989) is an example of such a study. However, mitomycin C is toxic, not only to hypoxic, but also to aerobic cells, and there is often little or no differential toxicity between hypoxic and aerobic cells (Fracasso & Sartorelli, 1986; Stratford & Stephens, 1989). Also, the drug was given only once during the course of the 6-week radiotherapy regimen, which, on theoretical grounds, would not be expected to modify the response compared to radiation alone if only hypoxic cells were killed (Brown & Koong, 1991). Thus, although the positive result of the combination compared to radiotherapy

**Figure 4** A model to account for the 'high LET' like damage caused in DNA by SR 4233 under hypoxia. The presence of an activating enzyme close to DNA would be expected to produce a high local concentration of activated SR 4233 radicals (shown emerging from the active site of the enzyme), each of which can produce a single strand break in the DNA. Also shown are representations of the ionisation densities in the tracks of a low and high LET ionising particles.

alone is encouraging, it is unlikely that this was the result of preferential killing of radiobiologically resistant hypoxic cells.

Two ways in which a bioreductive drug such as SR 4233 could be given clinically have been identified from preclinical studies. These are outlined below:

(a) *In combination with an agent which increases tumour hypoxia* It has been known for a number of years that certain vasoactive drugs can produce selective tumour hypoxia (Cater *et al.*, 1962; Voorhees & Babbs, 1982). Such hypoxia is a result of decreased tumour blood flow which, if prolonged, can produce cell killing in its own right. This appears to be the major mechanism for the antitumour action of flavone acetic acid (Zwi *et al.*, 1989). It might be predicted, then, that enhanced tumour cytotoxicity would be produced by combining one of these agents with a bioreductive cytotoxic agent. Results supporting this hypothesis have in fact been obtained for a combination of the vasodilating drug, hydralazine, with RSU 1069 (Chaplin & Acker, 1987; Bremner *et al.*, 1990), or with SR 4233 (Brown, 1987). SR 4233 has also been shown to enhance the antitumour effect of flavone acetic acid in murine tumours (Sun & Brown, 1989).

Thus, it would appear from these preclinical data that SR 4233, or other bioreductive cytotoxic drugs, could be effectively used with any agent that selectively increases hypoxia in human tumours. Unfortunately, it is yet to be demonstrated that decreased tumour blood flow and/or increased hypoxia can be produced in human tumours. Nonetheless, this remains a viable strategy for the use of a bioreductive drug.

**Figure 5** Response of SCCVII tumours to SR 4233 alone (0.11 mmol/kg/injection), to X-rays alone (2.5 Gy/fraction every 12 h) and a combination of the two (SR 4233 given 30 min before each radiation dose). The mice were sacrificed and the tumours removed to obtain clonogenic cell survival 12 h after the last dose. The lines are best fit least squares regression lines constrained to pass through 1 at zero dose. The line marked 'Additive' shows the product of the survivals for X-rays and for SR 4233 alone. Pooled data from two experiments. Redrawn from Brown and Lemmon (1990) with permission of the publisher.

(b) *In combination with fractionated irradiation* A theoretical study by Brown and Koong has suggested that hypoxia in tumours can be an advantage to radiation therapy if an agent specifically toxic to hypoxic cells can be given with most of the radiation doses (Brown & Koong, 1991). They demonstrated that a hypoxic cell cytotoxin could produce more tumour cell killing (by several orders of magnitude) than that produced by full oxygenation of the tumour or by use of an optimum hypoxic cell radiosensitiser, provided the cytotoxin kills more than ~50% of the hypoxic cells each time it is given. Experimental verification of this concept has come from studies combining SR 4233 with fractionated irradiation of mouse tumours (Brown & Lemmon, 1990). Essentially, the authors found, using both growth delay and cell survival, that it was more effective to combine SR 4233, rather than a massive dose of the radiation sensitiser, SR 2508, with each radiation dose in a multifraction regimen. Figure 5 shows data from this study demonstrating that SR 4233 produces an apparently greater than additive response when combined with fractionated irradiation. However, the calculated 'additive' response is misleading in that it assumes a homogenous cell population in the tumour. If it is assumed that the tumour has aerobic cells preferentially killed by ionising radiation and hypoxic cells preferentially killed by SR 4233, then a response essentially identical to that observed experimentally is predicted (Brown & Lemmon, 1990).

This enhancement of the antitumour effect with fractionated irradiation has been shown to be specific for the tumour, as there was no enhancement of early skin reactions (Brown & Lemmon, 1991*b*) or late reactions (Brown & Lemmon, 1991*a*) produced when SR 4233 was combined with fractionated irradiation.

*Is there potential to combine SR 4233 with chemotherpy?*

Although the data from clinical studies on the role of tumour hypoxia are not as compelling for chemotherapeutic agents as they are for ionising radiation, there is nonetheless evidence from preclinical studies that the hypoxic cells in solid tumours are refractory to certain chemotherapy drugs either because of their intrinsic hypoxia, their distance from blood vessels, their low repair capacity or their slower rate of proliferation (Kennedy, 1987; Sartorelli, 1988; Tannock & Guttman, 1981). If this is the case, then an improvement in the therapeutic index might be expected by combining the chemotherapeutic agent with a drug selectivity toxic to hypoxic cells. This was the initial rationale for the combination of the nitroimidazole hypoxic cytotoxic agents with various anticancer drugs, and results with one drug, doxorubicin, demonstrated that this rationale appeared to hold (Tannock, 1980).

This success encouraged several investigators to study nitroimidazole/anticancer drug interactions, and it soon became obvious that the nitroimidazoles could produce a therapeutic gain when combined with various anticancer agents, particularly the alkylating agents cyclophosphamide and melphalan, and the nitrosoureas BCNU and CCNU (Siemann, 1984). However, it became clear that the therapeutic gain produced by these agents was not the consequence of selective killing of hypoxic cells, but of other mechanisms including a potentiation of DNA crosslink formation by breakdown products of the nitroimidazoles (Taylor *et al.*, 1983).

With the advent of more potent bioreductive drugs than the nitroimidazoles (e.g., SR 4233 and RB 6145), there is now an opportunity for the first time to test in a rigorous manner the hypothesis that selective targeting of the hypoxic cells in solid tumours can enhance the effectiveness of certain anticancer drugs. Early studies with SR 4233 by Holden and colleagues (Holden *et al.*, 1992) are encouraging in this regard. These investigators found that they were able to markedly potentiate the tumour cell killing of cisplatin, melphalan, cyclophosphamide and BCNU when the tumour-bearing mice were given a large single dose of SR 4233 at the same time as the anticancer drug. Although the authors also observed some potentiation of bone marrow toxicity, they found a therapeutic gain for each of these antitumour agents. Recent studies by Dorie and Brown (unpublished) have also demonstrated that SR 4233 produces a large, schedule-dependent enhancement of tumour cell kill by cisplatin. These data encourage further studies, particularly as they raise a number of important questions. For example, it is not clear that the potentiation of tumour cell killing by the anticancer drugs is the result of a selective killing of chemotherapy resistant hypoxic cells. Studies to investigate the mechanism and clinical potential of this approach are clearly required.

*Can SR 4233 be given in sufficiently high doses with daily radiotherapy?*

As mentioned previously, theoretical studies of the combination of radiation with a hypoxic cytotoxic agent suggests that for optimum enhancement of tumour cell kill, the drug is best given with each radiation dose (Brown & Koong, 1991). Toxicological studies have been performed giving the drug daily (5 × per week) for 6 weeks to mice at doses which produce substantial enhancement of fractionated irradiation (Spiegel *et al.*, 1993). These authors found that such a dose was well-tolerated by the mice, producing no weight loss and no observable histopathological toxicity, but reduced peripheral white cell count to a steady level of approximately 30% of normal during the 6 weeks of injections. This white cell depression, plus a 20% drop in hematocrit, led the authors to suggest that the dose-limiting toxicity for daily injections of SR 4233 would be myelosuppression.

In assessing whether tumour cell cytotoxic doses can be given daily to man, it is necessary to know whether the cytotoxicity to hypoxic cells depends on area under the curve (AUC) of drug concentration × time. This has been established for hamster cells exposed *in vitro* for periods of from 20 min to 4 h (Tosto & Brown, unpublished). Assuming that this is the case *in vivo*, then toxicological studies already performed in dogs show that AUC values sufficient to produce hypoxic cell cytotoxicity can be achieved with nontoxic daily doses of SR 4233 (Hincks *et al.*, personal communication). Thus, knowledge of maximum tolerated doses and the pharmacokinetics of SR 4233 from currently ongoing Phase 1 studies will determine whether efficacious doses will be possible on a daily dose basis in man. Assuming values close to these are achievable, then this will provide substantial encouragement to further work with SR 4233 or its analogs.

### Unanswered questions

Major unanswered questions for the biological activity of SR 4233 and analogs are listed below:

(a) *The enzyme(s) responsible for metabolism of SR 4233 close to DNA* Apart from the obvious interest of determining these enzymes, the practical use of an assay for such nuclear localised enzymes would be to determine whether the *in vivo* efficacy for individual human solid tumours could be predicted. The greater the activity of such enzyme in an individual tumour, the more sensitive one would expect the tumour to be to killing by SR 4233 under hypoxia.

(b) *The mechanism, and critical cellular target, for the toxicity of SR 4233 to cells under aerobic conditions* From Figure 1, showing the mechanism for hypoxic cytotoxicity, it is apparent that the aerobic toxicity could be the result of the parent compound, the superoxide anion radical or the drug radical. No convincing evidence exists to decide among these three possibilities. Also unknown is the cellular target damage to which is primarily responsible for killing of cells under aerobic conditions. An important application of such knowledge would be that if systemic toxicity was related to aerobic toxicity and if aerobic and hypoxic toxicity had different mechanisms or target molecules, then it might be possible to increase tumour cell kill without changing systemic toxicity or decrease systemic toxicity without changing hypoxic toxicity.

(c) *Mechanism for bone marrow toxicity* in vivo It is not clear why bone marrow cells are more susceptible to the aerobic toxicity of SR 4233, and, if so, which blood precursor cell is most sensitive. Again, this may help to alleviate the systemic toxicity of the drug.

### Closing remarks

Preclinical studies and theoretical modeling have demonstrated that the combination of SR 4233 with fractionated irradiation of a solid tumour can produce several orders of magnitude more cell kill than that produced by radiation alone. It appears that this strategy is superior to the use of hypoxic cell radiosensitisers or agents which can oxygenate the hypoxic cells. Such an effect depends on the solid tumour containing hypoxic cells which are regenerated during therapy, a process analogous to reoxygenation during fractionated radiotherapy which we have termed 'rehypoxiation'. We know, at least, that a sizeable fraction (perhaps 40 to 50%) of human tumours contain hypoxic cells, but not whether the phenomenon of rehypoxiation occurs in human tumours. However, if human tumours behave in a similar manner to rodent tumours in this regard and if the human is not especially sensitive to the systemic toxicity of SR 4233 compared to mice and dogs, then substantial extra cell killing of the cells of solid tumours should be obtainable with SR 4233 combined with fractionated radiotherapy. The extent of this extra cell killing should lead to substantial increases in local control rates. These considerations qualify SR 4233 as the lead compound in an interesting new series of bioreductive drugs of clinical potential.

This study was supported by grant CA 15201 awarded by the US National Cancer Institute, D.H.H.S. The author would like to express his appreciation to his many laboratory colleagues who over the years have obtained much of the data upon which this review is based. They include M. Baker, K. Biedermann, I.L. Kim, M. Lemmon, P. McAfee, J. Sun, J. Wang and E. Zeman. The author would also like to thank Ms Chiyoye Adachi for her secretarial help with this paper.

### References

ADAMS, G.E. (1981). Hypoxia-mediated drugs for radiation and chemotherapy. *Cancer*, **48**, 696–707.

ADAMS, G.E., STRATFORD, I.J., EDWARDS, H.S., BREMNER, J.C.M. & COLES, S. (1992). Bioreductive drugs as post-irradiation sensitizers: comparison of dual function agents with SR 4233 and the mitomycin C analogue E09. *Int. J. Radiat. Oncol. Biol. Phys.*, **22**, 717–720.

BAKER, M.A., ZEMAN, E.M., HIRST, V.K. & BROWN, J.M. (1988). Metabolism of SR 4233 by Chinese hamster ovary cells: basis of selective hypoxic cytotoxicity. *Cancer Res.*, **48**, 5947–5952.

BEDFORD, J.S. & GOODHEAD, D.T. (1989). Breakage of human interphase chromosomes by α particles and X-rays. *Int. J. Radiat. Biol.*, **55**, 211–216.

BIEDERMANN, K.A., WANG, J., GRAHAM, R.P. & BROWN, J.M. (1991). SR 4233 cytotoxicity and metabolism in DNA repair-competent and repair-deficient cell cultures. *Br. J. Cancer*, **63**, 358–362.

BREMNER, J.C.M., STRATFORD, I.J., BOWLER, J. & ADAMS, G.E. (1990). Bioreductive drugs and the selective induction of tumour hypoxia. *Br. J. Cancer*, **61**, 717–721.

BROWN, J.M. (1987). Exploitation of bioreductive agents with vasoactive drugs. In *Radiation Research: Proceedings of the 8th International Congress of Radiation Research*, Fielden, E.M., Fowler, J.F., Hendry, J.H. & Scott, D. (eds). pp. 719–724. Taylor & Francis: London.

BROWN, J.M. (1989). Hypoxic cell radiosensitizers. Where next? *Int. J. Radiat. Oncol. Biol. Phys.*, **16**, 987–993.

BROWN, J.M. (1991). Redox activation of benzotriazine N-oxides: mechanisms and potential as anticancer drugs. In *Selective Activation of Drugs by Redox Processes*, Breccia, A., Adams, G.E., Fielden, E.M. & Wardman, P. (eds). pp. 137–148. Plenum Press: Fermo, Italy.

BROWN, J.M. & KOONG, A. (1991). Therapeutic advantage of hypoxic cells in tumors: a theoretical study. *JNCI*, **83**, 178–185.

BROWN, J.M. & LEMMON, M.J. (1990). Potentation by the hypoxic cytotoxin SR 4233 of cell killing produced by fractionated irradiation of mouse tumors. *Cancer Res.*, **50**, 7745–7749.

BROWN, J.M. & LEMMON, M.J. (1991*a*). SR 4233: a tumor specific radiosensitizer active in fractionated radiation regimens. *Radiother. & Oncol.*, **20**, 151–156.

BROWN, J.M. & LEMMON, M.J. (1991*b*). Tumor hypoxia can be exploited to preferentially sensitize tumors to fractionated irradiation. *Int. J. Radiat. Oncol. Biol. Phys.*, **20**, 457–461.

BROWN, J.M. & LEMMON, M.J. (1992). Fractionation increases the antitumor effect of adding a hypoxic cytotoxin to irradiation. In *Radiation Research a Twentieth Century Perspective, Volume II*, Dewey, W.C., Edington, M., Fry, R.J.M., Hall, E.J. & Whitmore, G.F. (eds). pp. 807–812. Academic Press: San Diego.

BUSH, R.S., JENKIN, R.D.T., ALLT, W.E.C., BEALE, F.A., BEAN, H., DENBO, A.J. & PRINGLE, J.F. (1978). Definite evidence for hypoxic cells influencing cure in cancer therapy. *Br. J. Cancer*, **37**, Suppl. III, 302–306.

CAHILL, A. & WHITE, I.N.H. (1990). Reductive metabolism of 3-amino-1,2,4-benzotriazine-1,4-dioxide (SR 4233) and the induction of unscheduled DNA synthesis in rat and human derived cell lines. *Carcinog.*, **11**, 1407–1411.

CATER, D.B., GRIGSON, C.M.B. & WATKINSON, D.A. (1962). Changes of oxygen tension in tumors induced by vasoconstrictor and vasodilator drugs. *Acta Radiol.*, **58**, 401–408.

CHAPLIN, D.J. & ACKER, B. (1987). The effect of hydralazine on the tumor cytotoxicity of the hypoxic cell cytotoxin RSU-1069: evidence for therapeutic gain. *Int. J. Radiat. Oncol. Biol. Phys.*, **13**, 579–585.

CHAPLIN, D.J., HORSMAN, M.R. & AOKI, D.S. (1991). Nicotinamide, Fluosol DNA and Carbogen: a strategy to reoxygenate acutely and chronically hypoxic cells *in vivo*. *Br. J. Cancer*, **63**, 109–113.

CORNFORTH, M.N. & BEDFORD, J.S. (1987). A quantitative comparison of potentially lethal damage repair and the rejoining of interphase chromosome breaks in low passage normal human fibroblasts. *Radiat. Res.*, **111**, 385–405.

COSTA, A.K., BAKER, M.A., BROWN, J.M. & TRUDELL, J.R. (1989). *In vitro* hepatotoxicity of SR 4233 (3-amino-1,2,4-benzotriazine-1,4-dioxide), a hypoxic cytotoxin and potential antitumor agent. *Cancer Res.*, **49**, 925–929.

FRACASSO, P.M. & SARTORELLI, A.C. (1986). Cytotoxicity and DNA lesions produced by mitomycin C and porfiromycin in hypoxic and aerobic EMT6 and Chinese hamster ovary cells. *Cancer Res.*, **46**, 3939–3944.

GATENBY, R.A., KESSLER, H.B., ROSENBLUM, J.S., COIA, L.R., MOLDOFSKY, P.J., HARTZ, W.H. & BRODER, G.J. (1988). Oxygen distribution in squamous cell carcinoma metastases and its relationship to outcome of radiation therapy. *Int. J. Radiat. Oncol. Biol. Phys.*, **14**, 831–838.

HENK, J.M. & SMITH, C.W. (1977). Radiotherapy and hyperbaric oxygen in head and neck cancer: interim report of second clinical trial. *Lancet*, **2**, 104.

HOCKEL, M., SCHLENGER, K., KNOOP, C. & VAUPEL, P. (1991). Oxygenation of carcinomas of the uterine cervix: evaluation by computerized $O_2$ tension measurements. *Cancer Res.*, **51**, 6098–6102.

HOLDEN, S.A., TEICHER, B.A., ARA, G., HERMAN, T.S. & COLEMAN, C.N. (1992). Enhancement of alkylating agent activity of SR-4233 in the FSaIIC murine fibrosarcoma. *JNCI*, **84**, 187–193.

JENKINS, T.C., NAYLOR, M.A., O'NEILL, P., THREADGILL, M.D., COLE, S., STRATFORD, I.J., ADAMS, G.E., FIELDEN, E.M., SUTO, M.J. & STIER, M.A. (1990). Synthesis and evaluation of: α[(2-Haloethyl)amino]methyl]-2-nitro-1-imidazole-1-ethanols as Prodrugs of α[(1-Aziridinyl)methyl]-2-nitro-1 H-imidazole-1-ethanol (RSU 1069) and its analogues which are radiosensitizers and bioreductively activated cytotoxins. *J. Med. Chem.*, **33**, 2603–2610.

KENNEDY, K.A. (1987). Hypoxic cells as specific drug targets for chemotherapy. *Anticancer Drug Des.*, **2**, 181–194.

KEOHANE, A., GODDEN, J., STRATFORD, I.J. & ADAMS, G.E. (1990). The effects of three bioreductive drugs (mitomycin C, RSU-1069 and SR 4233) on cell lines selected for their sensitivity to mitomycin C or ionising radiation. *Br. J. Cancer*, **61**, 722–726.

KIM, I.L. & BROWN, J.M. (1993). Reoxygenation and rehypoxiation in the SCCVII mouse tumor, 41st Annual Meeting of Radiation Research Society, Dallas, TX, March 19–25 (Abstr. p.31-21).

KJELLEN, E., JOINER, M.C., COLLIER, J.M., JOHNS, H. & ROJAS, A. (1991). A therapeutic benefit from combining normobaric carbogen or oxygen with nicotinamide in fractionated X-ray treatments. *Radiother. & Oncol.*, **22**, 81–91.

KOCH, C.J. (1984). A thin-film culturing technique allowing rapid gas-liquid equilibration (6 sec) with no toxicity to mammalian cells. *Radiat. Res.*, **97**, 434–442.

KOCH, C.J. (1990). A reductive activation of nitroimidazoles: modification by oxygen and other redox active molecules in cellular systems. In *Selective Activation of Drugs by Redox Processes*, Breccia, A., Adams, G.E., Fielden, E.M. & Wardman, P. (eds), pp. 237–247. Plenum Press: Fermo, Italy.

KOCH, C.J. (1991). Polarographic oxygen sensor. U.S. Patent 5030036. July, 1991.

KOCH, C.J. (1993). The unusual dependence on oxygen concentration of toxicity by SR 4233 [3-amino-1,2,4-benzotriazine 1,4-dioxide]: an hypoxic cell toxin. *Cancer Res.* (submitted).

LADEROUTE, K., WARDMAN, P. & RAUTH, A.M. (1988). Molecular mechanisms for the hypoxia-dependent activation of 3-amino-1,2,4-benzotriazine 1,4-dioxide (SR 4233). *Biochem. Pharmacol.*, **37**, 1487–1495.

LIN, A.J., COSBY, L.A. & SARTORELLI, A.C. (1976). Potential bioreductive alkylating agents. In *Cancer Chemotherapy*, Sartorelli, A.C. (ed.), pp. 71–86. American Chemical Society: Washington.

LIN, A.J., COSBY, L.A., SHANSKY, C.W. & SARTORELLI, A.C. (1972). Potential bioreductive alkyalting agents. 1. Benzoquinone derivatives. *J. Med. Chem.*, **15**, 1247–1252.

LLOYD, R.V., DULING, D.R., RUMYANTSEVA, G.V., MASON, R.P. & BRIDSON, P.K. (1991). Microsomal reduction of 3-amino-1,2,4-benzotriazine 1,4-dioxide to a free radical. *Mol. Pharmacol.*, **40**, 440–445.

MARSHALL, R.S. & RAUTH, A.M. (1986). Modification of the cytotoxic activity of mitomycin C by oxygen and ascorbic acid in Chinese hamster ovary cells and a repair-deficient mutant. *Cancer Res.*, **46**, 2709–2713.

MOORE, H.W. (1977). Bioactiviation as a model for drug design bioreductive alkylation. *Science*, **197**, 527–532.

MOULDER, J.E. & ROCKWELL, S. (1987). Tumor hypoxia: its impact on cancer therapy. *Cancer Metast. Rev.*, **5**, 313–341.

MUELLER-KLIESER, W., VAUPEL, P., MANZ, R. & SCHMIDSEDER, R. (1981). Intracapillary oxyhemoglobin saturation of malignant tumors in humans. *Int. J. Radiat. Onc. Biol. Phys.*, **7**, 1397–1404.

MULCAHY, R.T. (1984). Effect of oxygen on misonidazole chemosensitization and cytotoxicity *in vitro*. *Cancer Res.*, **44**, 4409–4413.

OVERGAARD, J., HANSEN, H.S., JORGENSEN, K. & HANSEN, M.H. (1986). Primary radiotherapy of larynx and pharynx carcinomas – an analysis of some factors influencing local control and survival. *Int. J. Radiat. Onc. Biol. Phys.*, **12**, 515–521.

PRISE, K.M., DAVIES, S. & MICHAEL, B.D. (1987). The relationship between radiation-induced DNA double-strand breaks and cell kill in hamster V79 fibroblasts irradiated with 250 kVp X-rays, 2.3 MeV neutrons or $^{238}$Pu a-particles. *Int. J. Radiat. Biol.*, **52**, 893–902.

REVELL, S.H. (1983). Relationship between chromosome damage and cell death. In *Radiation-Induced Chromosome Damage in Man*, Ishihara, T. & Sasaki, M.S. (eds), pp. 215–233. Alan R. Liss, Inc.: New York, NY.

ROCKWELL, S. & KENNEDY, K.A. (1979). Combination therapy with radiation and mitomycin C: preliminary results with EMT6 tumor cells *in vitro* and *in vivo*. *Int. J. Radiat. Oncol. Biol. Phys.*, **5**, 1673–1676.

ROCKWELL, S., KENNEDY, K.A. & SARTORELLI, A.C. (1982). Mitomycin-C as a prototype bioreductive alkylating agent: *in vitro* studies of metabolism and cytotoxicity. *Int. J. Radiat. Oncol. Biol. Phys.*, **8**, 753–755.

ROCKWELL, S. & MOULDER, J.E. (1990). Hypoxic fractions of human tumors xenografted into mice: a review. *Int. J. Radiat. Onc. Biol. Phys.*, **19**, 197–202.

ROIZIN-TOWLE, L., PIRRO, J.P. & HALL, E.J. (1990). Studies with bifunctional bioreductive drugs II. Cytotoxicity assayed with A-549 lung carcinoma cells of human origin. *Radiat. Res.*, **124**, S50–S55.

SARTORELLI, A.C. (1988). Therapeutic attack of hypoxic cells of solid tumors: presidential address. *Cancer Res.*, **48**, 775–778.

SIEMANN, D.W. (1984). Modification of chemotherapy by nitroimidazoles. *Int. J. Radiat. Oncol. Biol. Phys.*, **10**, 1585–1594.

SPIEGEL, J.F., SPEAR, M.A. & BROWN, J.M. (1993). Toxicology of daily administration to mice of the radiation potentiator SR 4233 (WIN 59075). *Radiotherapy & Oncol.*, **26**, 79–81.

STRATFORD, I.J. & STEPHENS, M.A. (1989). The differential hypoxic cytotoxicity of bioreductive agents determined *in vitro* by the MTT assay. *Int. J. Radiat. Oncol. Biol. Phys.*, **16**, 973–976.

STRATFORD, I.J., O'NEILL, P., SHELDON, P.W., SILVER, A.R.J., WALLING, J.M. & ADAMS, G.E. (1986). RSU 1069, A nitroimidazole containing an aziridine group – bioreduction greatly increases cytotoxicity under hypoxic conditions. *Biochem. Pharm.*, **35**, 105–109.

SUN, J.R. & BROWN, J.M. (1989). Enhancement of the antitumor effect of flavone acetic acid by the bioreductive cytotoxic drug SR 4233 in a murine carcinoma. *Cancer Res.*, **49**, 5664–5670.

TANNOCK, I. & GUTTMAN, P. (1981). Response of Chinese hamster ovary cells to anticancer drugs under aerobic and hypoxic conditions. *Br. J. Cancer*, **42**, 245–248.

TANNOCK, I.F. (1980). *In vivo* interaction of anti-cancer drugs with misonidazole or metronidazole: methotrexate, 5-fluorouracil and adriamycin. *Br. J. Cancer*, **42**, 861–870.

TAYLOR, Y.C., EVANS, J.W. & BROWN, J.M. (1983). Mechanism of sensitization of Chinese hamster ovary cells to melphalan by hypoxic treatment with misonidazole. *Cancer Res.*, **43**, 3175–3181.

TAYLOR, Y.C. & RAUTH, A.M. (1978). Differences in the toxicity and metabolism of the 2-nitroimidazole misonidazole (Ro-07-0582) in HeLa and Chinese hamster ovary cells. *Cancer Res.*, **38**, 2745–2752.

VAUPEL, P., SCHLENGER, K., KNOOP, C. & HOCKEL, M. (1991). Oxygenation of human tumors: evaluation of tissue oxygen distribution in breast cancers by computerized O2 tension measurements. *Cancer Res.*, **51**, 3316–3322.

VOORHEES, W.D. III & BABBS, C.F. (1982). Hydralazine-enhanced selective heating of transmissible venereal tumor implants in dogs. *Eur. J. Cancer Clin. Oncol.*, **18**, 1027–1033.

WALTON, M.I., WOLF, C.R. & WORKMAN, P. (1992). The role of cytochrome P450 and cytochrome P450 reductase in the reductive bioactivation of the novel benzotriazine di-N-oxide hypoxic cytotoxin 3-amino-1,2,4-benzotriazine 1,4-dioxide (SR 4233, WIN 59075) by mouse liver. *Biochem. Pharmacol.*, **44**, 251–259.

WALTON, M.I. & WORKMAN, P. (1990). Enzymology of the reductive bioactivation of SR 4233. *Biochem. Pharmacol.*, **39**, 1735–1742.

WANG, J., BIEDERMANN, K.A. & BROWN, J.M. (1992). Repair of DNA and chromosome breaks in cells exposed to SR 4233 under hypoxia or to ionizing radiation. *Cancer Res.*, **52**, 4473–4477.

WANG, J., BIEDERMANN, K.A., WOLF, C.R. & BROWN, J.M. (1993). Metabolism of the bioreductive cytotoxin SR 4233 by tumor cells: enzymatic studies. *Br. J. Cancer*, **67**, 321–325.

WEISSBERG, J.B., SON, Y.H., PAPAC, R.J., SASAKI, C., FISCHER, D.B., LAWRENCE, R., ROCKWELL, S., SARTORELLI, A.C. & FISCHER, J.J. (1989). Randomized clinical trial of mitomycin C as an adjunct to radiotherapy in head and neck cancer. *Int. J. Radiat. Oncol. Biol. Phys.*, **17**, 3–9.

ZEMAN, E.M. & BROWN, J.M. (1989). Pre- and post-irradiation radiosensitization by SR 4233. *Int. J. Rad. Oncol. Biol. Phys.*, **16**, 967–971.

ZEMAN, E.M., BROWN, J.M., LEMMON, M.J., HIRST, V.K. & LEE, W.W. (1986). SR 4233: a new bioreductive agent with high selective toxicity for hypoxic mammalian cells. *Int. J. Radiat. Oncol. Biol. Phys.*, **12**, 1239–1242.

ZEMAN, E.M., HIRST, V.K., LEMMON, M.J. & BROWN, J.M. (1988). Enhancement of radiation-induced tumor cell killing by the hypoxic cell toxin SR 4233. *Radiother. Oncol.*, **12**, 209–218.

ZWI, L.J., BAGULEY, B.C., GAVIN, J.B. & WILSON, W.R. (1989). Blood flow failure as a major determinant in the antitumor action of flavone acetic acid (NSC 347512). *JNCI*, **81**, 1005–1013.


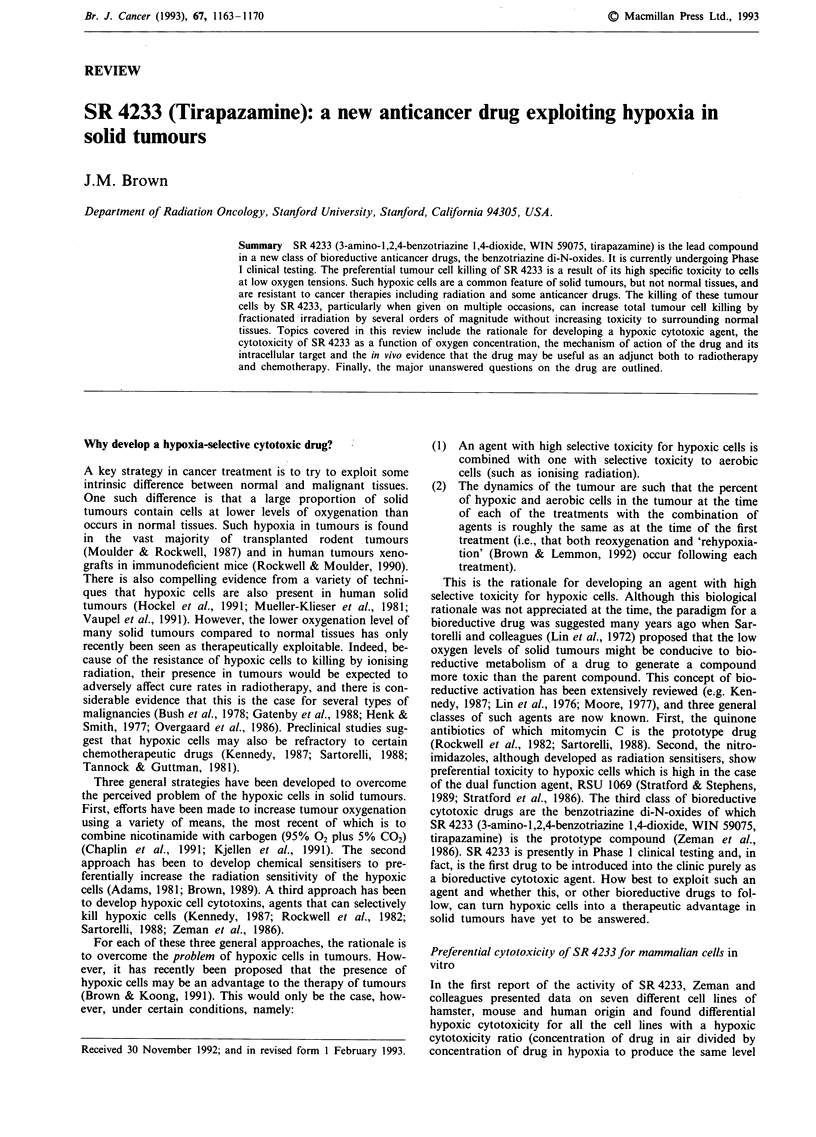

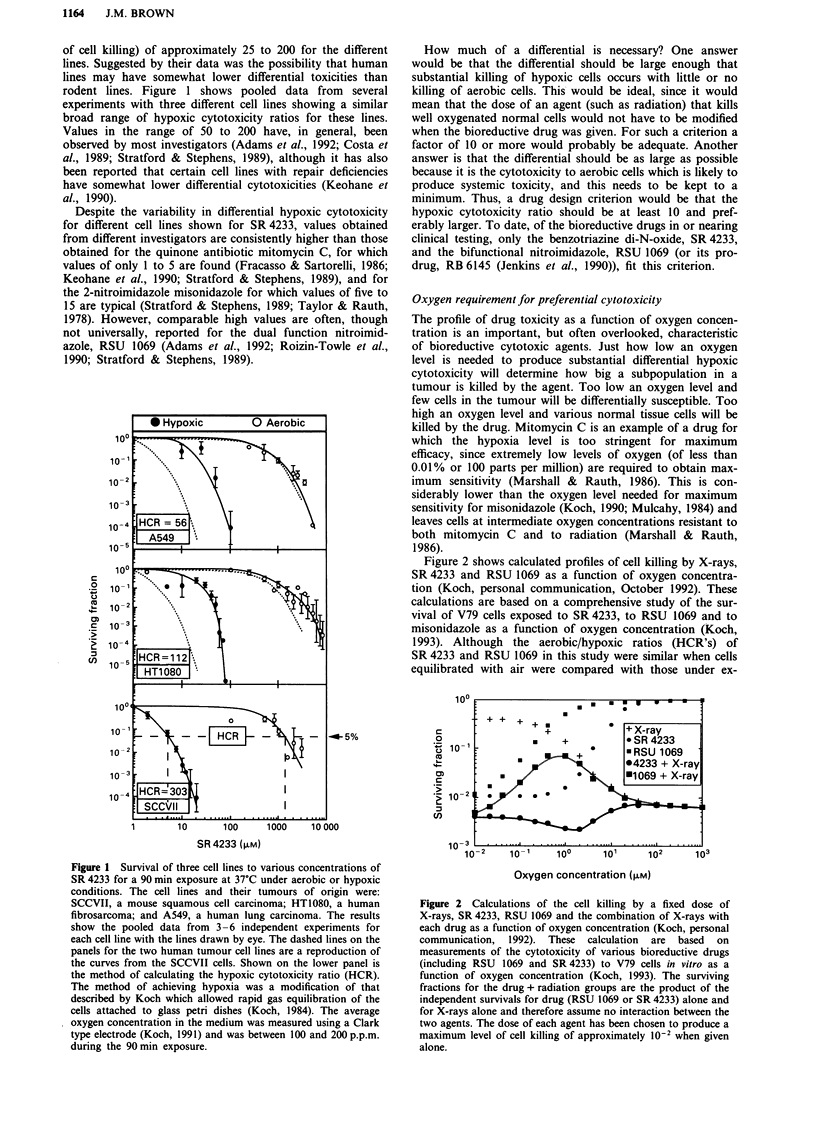

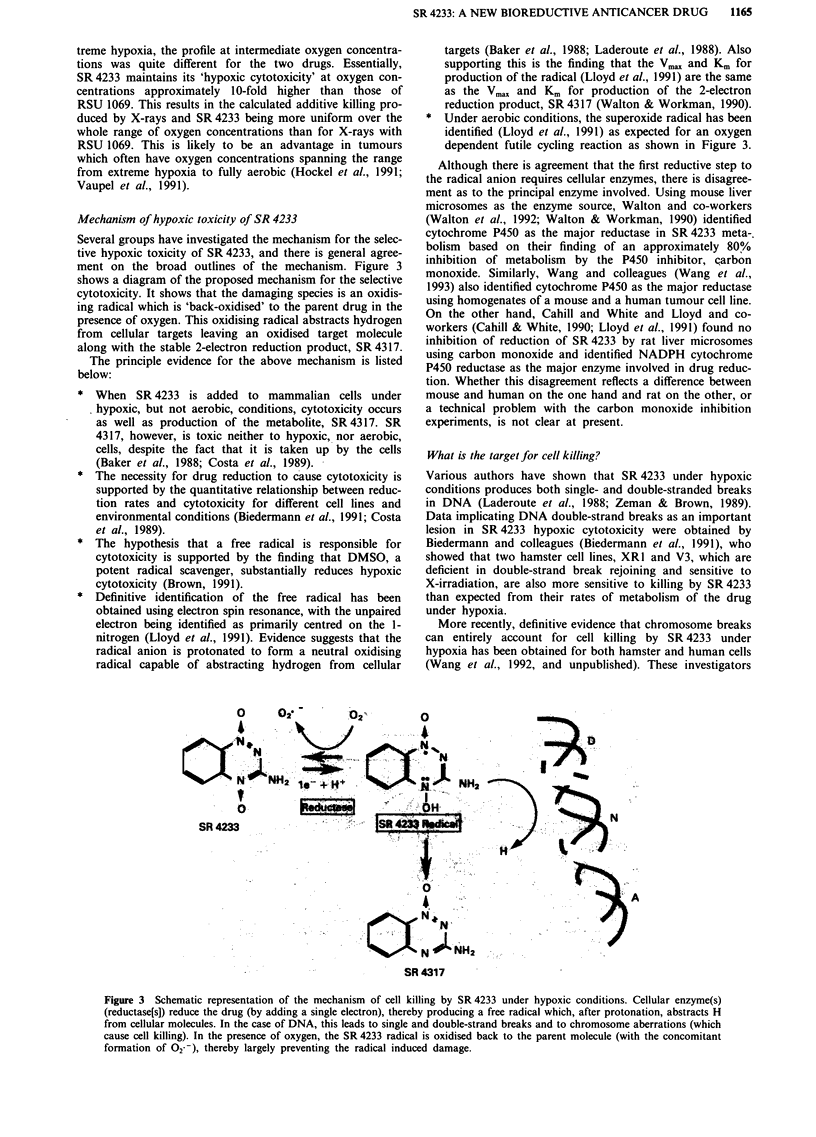

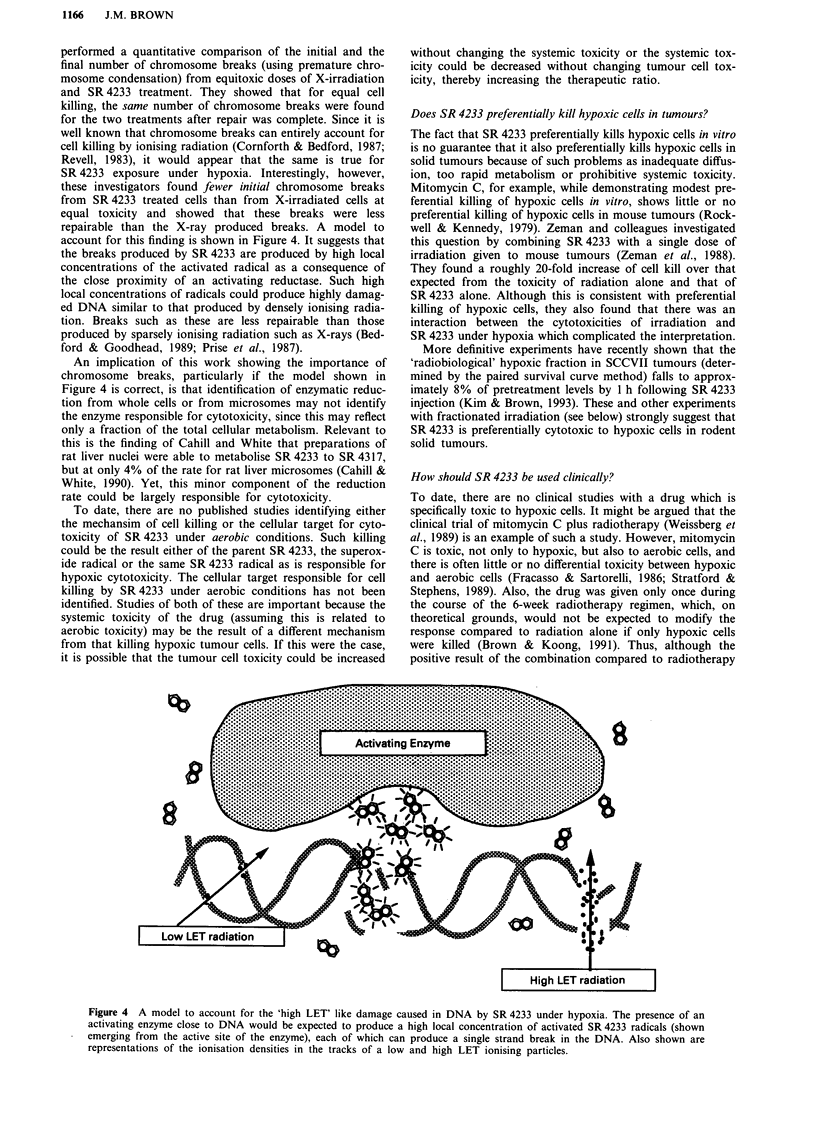

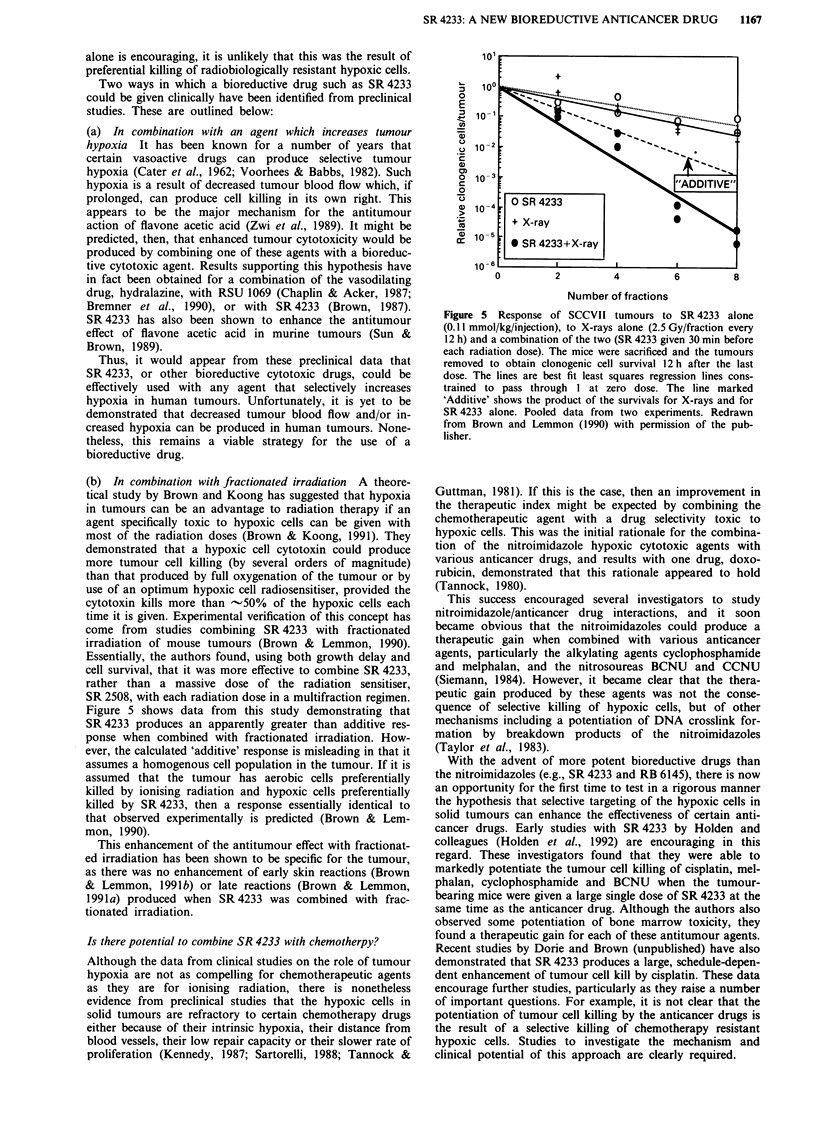

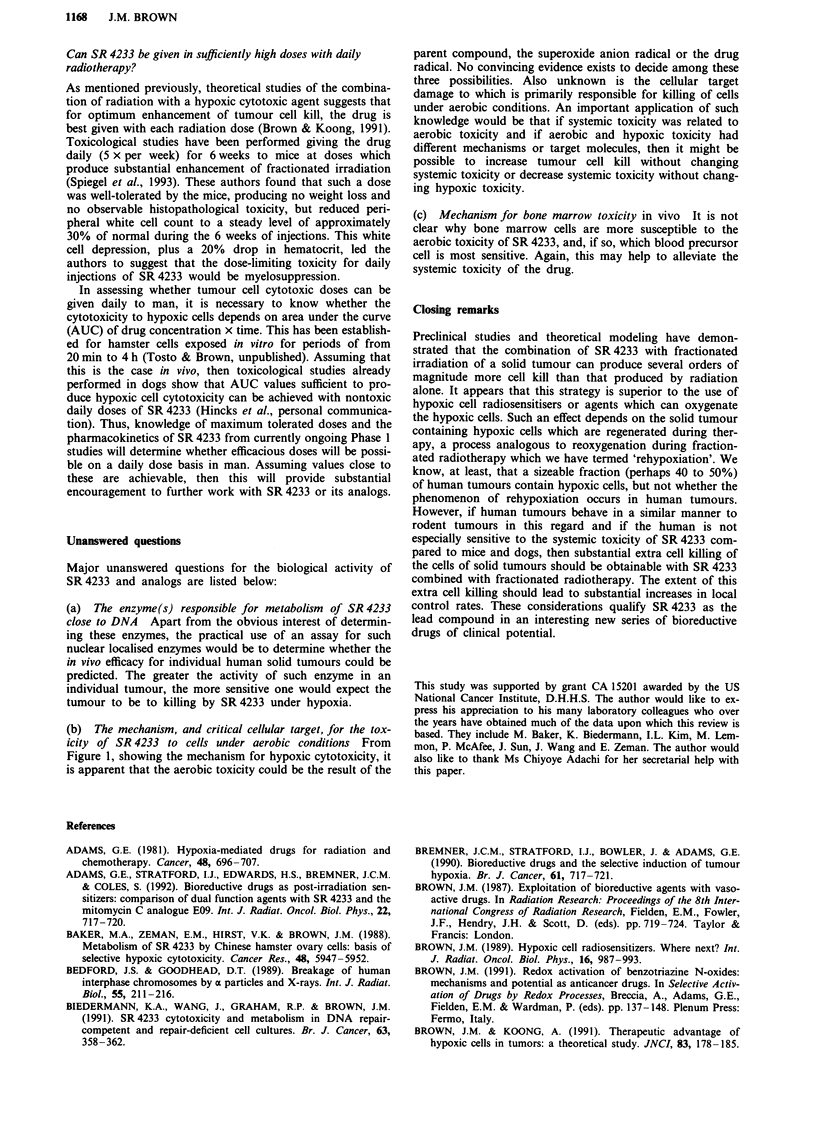

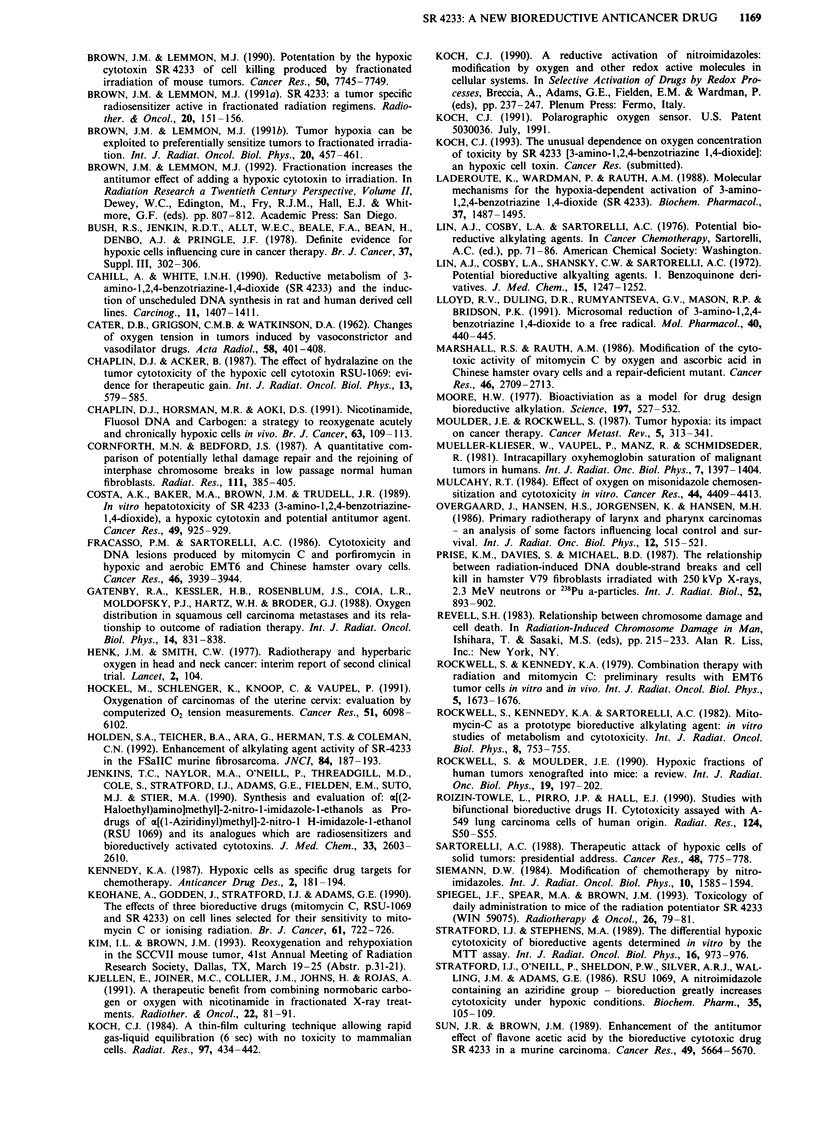

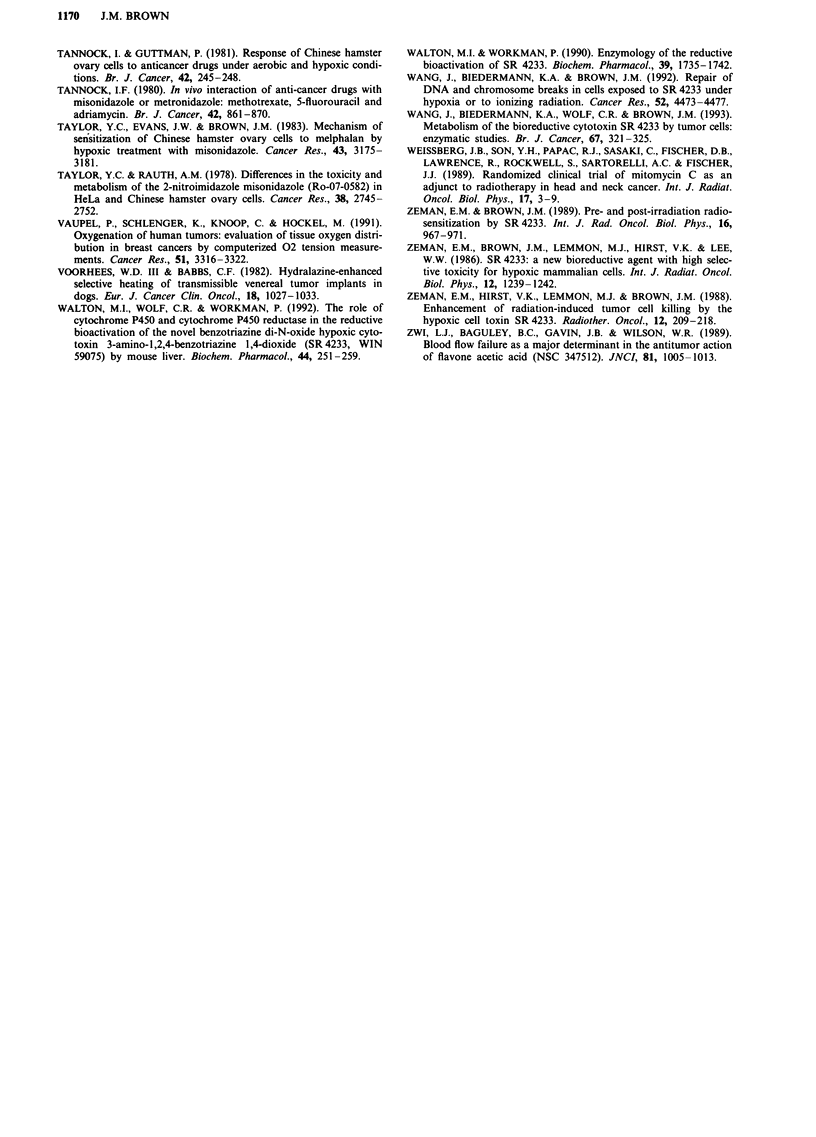


## References

[OCR_00798] Adams G. E. (1981). Hypoxia-mediated drugs for radiation and chemotherapy.. Cancer.

[OCR_00802] Adams G. E., Stratford I. J., Edwards H. S., Bremner J. C., Cole S. (1992). Bioreductive drugs as post-irradiation sensitizers: comparison of dual function agents with SR 4233 and the mitomycin C analogue EO9.. Int J Radiat Oncol Biol Phys.

[OCR_00809] Baker M. A., Zeman E. M., Hirst V. K., Brown J. M. (1988). Metabolism of SR 4233 by Chinese hamster ovary cells: basis of selective hypoxic cytotoxicity.. Cancer Res.

[OCR_00814] Bedford J. S., Goodhead D. T. (1989). Breakage of human interphase chromosomes by alpha particles and X-rays.. Int J Radiat Biol.

[OCR_00819] Biedermann K. A., Wang J., Graham R. P., Brown J. M. (1991). SR 4233 cytotoxicity and metabolism in DNA repair-competent and repair-deficient cell cultures.. Br J Cancer.

[OCR_00825] Bremner J. C., Stratford I. J., Bowler J., Adams G. E. (1990). Bioreductive drugs and the selective induction of tumour hypoxia.. Br J Cancer.

[OCR_00837] Brown J. M. (1989). Keynote address: hypoxic cell radiosensitizers: where next?. Int J Radiat Oncol Biol Phys.

[OCR_00848] Brown J. M., Koong A. (1991). Therapeutic advantage of hypoxic cells in tumors: a theoretical study.. J Natl Cancer Inst.

[OCR_00854] Brown J. M., Lemmon M. J. (1990). Potentiation by the hypoxic cytotoxin SR 4233 of cell killing produced by fractionated irradiation of mouse tumors.. Cancer Res.

[OCR_00859] Brown J. M., Lemmon M. J. (1991). SR 4233: a tumor specific radiosensitizer active in fractionated radiation regimes.. Radiother Oncol.

[OCR_00864] Brown J. M., Lemmon M. J. (1991). Tumor hypoxia can be exploited to preferentially sensitize tumors to fractionated irradiation.. Int J Radiat Oncol Biol Phys.

[OCR_00876] Bush R. S., Jenkin R. D., Allt W. E., Beale F. A., Bean H., Dembo A. J., Pringle J. F. (1978). Definitive evidence for hypoxic cells influencing cure in cancer therapy.. Br J Cancer Suppl.

[OCR_00888] CATER D. B., GRIGSON C. M., WATKINSON D. A. (1962). Changes of oxygen tension in tumours induced by vasoconstrictor and vasodilator drugs.. Acta radiol.

[OCR_00882] Cahill A., White I. N. (1990). Reductive metabolism of 3-amino-1,2,4-benzotriazine-1,4-dioxide (SR 4233) and the induction of unscheduled DNA synthesis in rat and human derived cell lines.. Carcinogenesis.

[OCR_00893] Chaplin D. J., Acker B. (1987). The effect of hydralazine on the tumor cytotoxicity of the hypoxic cell cytotoxin RSU-1069: evidence for therapeutic gain.. Int J Radiat Oncol Biol Phys.

[OCR_00899] Chaplin D. J., Horsman M. R., Aoki D. S. (1991). Nicotinamide, Fluosol DA and Carbogen: a strategy to reoxygenate acutely and chronically hypoxic cells in vivo.. Br J Cancer.

[OCR_00903] Cornforth M. N., Bedford J. S. (1987). A quantitative comparison of potentially lethal damage repair and the rejoining of interphase chromosome breaks in low passage normal human fibroblasts.. Radiat Res.

[OCR_00909] Costa A. K., Baker M. A., Brown J. M., Trudell J. R. (1989). In vitro hepatotoxicity of SR 4233 (3-amino-1,2,4-benzotriazine-1,4-dioxide), a hypoxic cytotoxin and potential antitumor agent.. Cancer Res.

[OCR_00915] Fracasso P. M., Sartorelli A. C. (1986). Cytotoxicity and DNA lesions produced by mitomycin C and porfiromycin in hypoxic and aerobic EMT6 and Chinese hamster ovary cells.. Cancer Res.

[OCR_00921] Gatenby R. A., Kessler H. B., Rosenblum J. S., Coia L. R., Moldofsky P. J., Hartz W. H., Broder G. J. (1988). Oxygen distribution in squamous cell carcinoma metastases and its relationship to outcome of radiation therapy.. Int J Radiat Oncol Biol Phys.

[OCR_00928] Henk J. M., Smith C. W. (1977). Radiotherapy and hyperbaric oxygen in head and neck cancer. Interim report of second clinical trial.. Lancet.

[OCR_00939] Holden S. A., Teicher B. A., Ara G., Herman T. S., Coleman C. N. (1992). Enhancement of alkylating agent activity by SR-4233 in the FSaIIC murine fibrosarcoma.. J Natl Cancer Inst.

[OCR_00933] Höckel M., Schlenger K., Knoop C., Vaupel P. (1991). Oxygenation of carcinomas of the uterine cervix: evaluation by computerized O2 tension measurements.. Cancer Res.

[OCR_00944] Jenkins T. C., Naylor M. A., O'Neill P., Threadgill M. D., Cole S., Stratford I. J., Adams G. E., Fielden E. M., Suto M. J., Stier M. A. (1990). Synthesis and evaluation of alpha-[[(2-haloethyl)amino]methyl]-2- nitro-1H-imidazole-1-ethanols as prodrugs of alpha-[(1-aziridinyl)methyl]-2- nitro-1H-imidazole-1-ethanol (RSU-1069) and its analogues which are radiosensitizers and bioreductively activated cytotoxins.. J Med Chem.

[OCR_00954] Kennedy K. A. (1987). Hypoxic cells as specific drug targets for chemotherapy.. Anticancer Drug Des.

[OCR_00958] Keohane A., Godden J., Stratford I. J., Adams G. E. (1990). The effects of three bioreductive drugs (mitomycin C, RSU-1069 and SR4233) on cell lines selected for their sensitivity to mitomycin C or ionising radiation.. Br J Cancer.

[OCR_00969] Kjellen E., Joiner M. C., Collier J. M., Johns H., Rojas A. (1991). A therapeutic benefit from combining normobaric carbogen or oxygen with nicotinamide in fractionated X-ray treatments.. Radiother Oncol.

[OCR_00975] Koch C. J. (1984). A thin-film culturing technique allowing rapid gas-liquid equilibration (6 sec) with no toxicity to mammalian cells.. Radiat Res.

[OCR_00996] Laderoute K., Wardman P., Rauth A. M. (1988). Molecular mechanisms for the hypoxia-dependent activation of 3-amino-1,2,4-benzotriazine-1,4-dioxide (SR 4233).. Biochem Pharmacol.

[OCR_01006] Lin A. J., Cosby L. A., Shansky C. W., Sartorelli A. C. (1972). Potential bioreductive alkylating agents. 1. Benzoquinone derivatives.. J Med Chem.

[OCR_01011] Lloyd R. V., Duling D. R., Rumyantseva G. V., Mason R. P., Bridson P. K. (1991). Microsomal reduction of 3-amino-1,2,4-benzotriazine 1,4-dioxide to a free radical.. Mol Pharmacol.

[OCR_01017] Marshall R. S., Rauth A. M. (1986). Modification of the cytotoxic activity of mitomycin C by oxygen and ascorbic acid in Chinese hamster ovary cells and a repair-deficient mutant.. Cancer Res.

[OCR_01023] Moore H. W. (1977). Bioactivation as a model for drug design bioreductive alkylation.. Science.

[OCR_01027] Moulder J. E., Rockwell S. (1987). Tumor hypoxia: its impact on cancer therapy.. Cancer Metastasis Rev.

[OCR_01031] Mueller-Klieser W., Vaupel P., Manz R., Schmidseder R. (1981). Intracapillary oxyhemoglobin saturation of malignant tumors in humans.. Int J Radiat Oncol Biol Phys.

[OCR_01035] Mulcahy R. T. (1984). Effect of oxygen on misonidazole chemosensitization and cytotoxicity in vitro.. Cancer Res.

[OCR_01038] Overgaard J., Hansen H. S., Jørgensen K., Hjelm Hansen M. (1986). Primary radiotherapy of larynx and pharynx carcinoma--an analysis of some factors influencing local control and survival.. Int J Radiat Oncol Biol Phys.

[OCR_01044] Prise K. M., Davies S., Michael B. D. (1987). The relationship between radiation-induced DNA double-strand breaks and cell kill in hamster V79 fibroblasts irradiated with 250 kVp X-rays, 2.3 MeV neutrons or 238Pu alpha-particles.. Int J Radiat Biol Relat Stud Phys Chem Med.

[OCR_01057] Rockwell S., Kennedy K. A. (1979). Combination therapy with radiation and mitomycin C: preliminary results with EMT6 tumor cells in vitro and in vivo.. Int J Radiat Oncol Biol Phys.

[OCR_01063] Rockwell S., Kennedy K. A., Sartorelli A. C. (1982). Mitomycin-C as a prototype bioreductive alkylating agent: in vitro studies of metabolism and cytotoxicity.. Int J Radiat Oncol Biol Phys.

[OCR_01069] Rockwell S., Moulder J. E. (1990). Hypoxic fractions of human tumors xenografted into mice: a review.. Int J Radiat Oncol Biol Phys.

[OCR_01074] Roizin-Towle L., Pirro J. P., Hall E. J. (1990). Studies with bifunctional bioreductive drugs. II. Cytotoxicity assayed with A-549 lung carcinoma cells of human origin.. Radiat Res.

[OCR_01080] Sartorelli A. C. (1988). Therapeutic attack of hypoxic cells of solid tumors: presidential address.. Cancer Res.

[OCR_01084] Siemann D. W. (1984). Modification of chemotherapy by nitroimidazoles.. Int J Radiat Oncol Biol Phys.

[OCR_01088] Spiegel J. F., Spear M. A., Brown J. M. (1993). Toxicology of daily administration to mice of the radiation potentiator SR 4233 (WIN 59075).. Radiother Oncol.

[OCR_01100] Stratford I. J., O'Neill P., Sheldon P. W., Silver A. R., Walling J. M., Adams G. E. (1986). RSU 1069, a nitroimidazole containing an aziridine group. Bioreduction greatly increases cytotoxicity under hypoxic conditions.. Biochem Pharmacol.

[OCR_01093] Stratford I. J., Stephens M. A. (1989). The differential hypoxic cytotoxicity of bioreductive agents determined in vitro by the MTT assay.. Int J Radiat Oncol Biol Phys.

[OCR_01105] Sun J. R., Brown J. M. (1989). Enhancement of the antitumor effect of flavone acetic acid by the bioreductive cytotoxic drug SR 4233 in a murine carcinoma.. Cancer Res.

[OCR_01117] Tannock I. F. (1980). In vivo interaction of anti-cancer drugs with misonidazole or metronidazole: methotrexate, 5-fluorouracil and adriamycin.. Br J Cancer.

[OCR_01112] Tannock I., Guttman P. (1981). Response of Chinese hamster ovary cells to anticancer drugs under aerobic and hypoxic conditions.. Br J Cancer.

[OCR_01122] Taylor Y. C., Evans J. W., Brown J. M. (1983). Mechanism of sensitization of Chinese hamster ovary cells to melphalan by hypoxic treatment with misonidazole.. Cancer Res.

[OCR_01128] Taylor Y. C., Rauth A. M. (1978). Differences in the toxicity and metabolism of the 2-nitroimidazole misonidazole (Ro-07-0582) in HeLa and Chinese hamster ovary cells.. Cancer Res.

[OCR_01134] Vaupel P., Schlenger K., Knoop C., Höckel M. (1991). Oxygenation of human tumors: evaluation of tissue oxygen distribution in breast cancers by computerized O2 tension measurements.. Cancer Res.

[OCR_01140] Voorhees W. D., Babbs C. F. (1982). Hydralazine-enhanced selective heating of transmissible venereal tumor implants in dogs.. Eur J Cancer Clin Oncol.

[OCR_01145] Walton M. I., Wolf C. R., Workman P. (1992). The role of cytochrome P450 and cytochrome P450 reductase in the reductive bioactivation of the novel benzotriazine di-N-oxide hypoxic cytotoxin 3-amino-1,2,4-benzotriazine-1,4-dioxide (SR 4233, WIN 59075) by mouse liver.. Biochem Pharmacol.

[OCR_01152] Walton M. I., Workman P. (1990). Enzymology of the reductive bioactivation of SR 4233. A novel benzotriazine di-N-oxide hypoxic cell cytotoxin.. Biochem Pharmacol.

[OCR_01155] Wang J., Biedermann K. A., Brown J. M. (1992). Repair of DNA and chromosome breaks in cells exposed to SR 4233 under hypoxia or to ionizing radiation.. Cancer Res.

[OCR_01160] Wang J., Biedermann K. A., Wolf C. R., Brown J. M. (1993). Metabolism of the bioreductive cytotoxin SR 4233 by tumour cells: enzymatic studies.. Br J Cancer.

[OCR_01165] Weissberg J. B., Son Y. H., Papac R. J., Sasaki C., Fischer D. B., Lawrence R., Rockwell S., Sartorelli A. C., Fischer J. J. (1989). Randomized clinical trial of mitomycin C as an adjunct to radiotherapy in head and neck cancer.. Int J Radiat Oncol Biol Phys.

[OCR_01177] Zeman E. M., Brown J. M., Lemmon M. J., Hirst V. K., Lee W. W. (1986). SR-4233: a new bioreductive agent with high selective toxicity for hypoxic mammalian cells.. Int J Radiat Oncol Biol Phys.

[OCR_01172] Zeman E. M., Brown J. M. (1989). Pre- and post-irradiation radiosensitization by SR 4233.. Int J Radiat Oncol Biol Phys.

[OCR_01183] Zeman E. M., Hirst V. K., Lemmon M. J., Brown J. M. (1988). Enhancement of radiation-induced tumor cell killing by the hypoxic cell toxin SR 4233.. Radiother Oncol.

[OCR_01188] Zwi L. J., Baguley B. C., Gavin J. B., Wilson W. R. (1989). Blood flow failure as a major determinant in the antitumor action of flavone acetic acid.. J Natl Cancer Inst.

